# Nuclear FAK and its kinase activity regulate VEGFR2 transcription in angiogenesis of adult mice

**DOI:** 10.1038/s41598-018-20930-z

**Published:** 2018-02-07

**Authors:** Shaogang Sun, Hsin-Jung Wu, Jun-Lin Guan

**Affiliations:** Departments of Cancer Biology, University of Cincinnati College of Medicine, Cincinnati, OH 45267 USA

## Abstract

Focal adhesion kinase (FAK) is essential in embryonic angiogenesis by regulating endothelial cell (EC) survival and barrier functions through its kinase-independent and –dependent activities. Here, we generated EC-specific tamoxifen-inducible FAK knockout and FAK kinase-defective (KD) mutant knockin mice to investigate the role of FAK and its kinase activity in angiogenesis of adult animals. Unlike previous observations of their differential defects in embryonic vascular development, both FAK ablation and inactivation of its kinase activity resulted in deficient angiogenesis in wound-healing as well as retinal angiogenesis models. Consistent with these phenotypes, loss of FAK or its kinase activity decreased EC proliferation and migration to similar extents, suggesting FAK primarily acts as a kinase for the regulation of adult EC-mediated angiogenesis. Further mechanistic analyses were carried out using an established mouse EC line MS1 cells. Interestingly, we found that FAK regulated the expression of VEGFR2, a central mediator of various EC functions and angiogenesis, which requires both FAK kinase activity and its translocation into the nucleus. Moreover, nuclear FAK was detected in the RNA polymerase II complex associated with VEGFR2 promoter, suggesting its direct participation in the transcriptional regulation of VEGFR2. Together, our results provide significant insights into the signaling mechanisms of FAK in angiogenesis that may contribute to future design of more effective angiogenesis related therapy.

## Introduction

Angiogenesis is a complex biological process which plays an essential role in embryogenesis, the homeostasis of adult animals, and various diseases including coronary heart disease, age-related macular degeneration, diabetes and cancer^1–5^. Endothelial cells (ECs) are central players in angiogenesis, and their responses to extracellular stimuli such as vascular endothelial growth factor (VEGF) are crucial in angiogenesis during embryogenesis and in adult organisms. Of several VEGF receptors, VEGFR2 has been identified as a principal mediator of various physiological and pathological effects of VEGF on ECs, including proliferation, migration, survival and permeability^6^.

Focal adhesion kinase (FAK) is a major mediator of signal transduction by integrins and also participates in signaling by growth factor receptors such as VEGF receptors in ECs^7–13^. Consistent with its roles in diverse cellular functions of various cells, FAK has been shown to regulate EC migration, proliferation and survival in previous studies. VEGFR2 activation by VEGF stimulates FAK phosphorylation, its localization to nascent focal adhesion, as well as its association with other focal adhesion and signaling molecules including paxillin and PI3-kinase, which are required for promoting EC migration^14^. In addition to the better characterized role of FAK in mediating signaling events by integrins and other receptors at the plasma membrane, recent studies also suggested nuclear translocation of FAK under certain conditions^15,16^, consistent with the presence of putative nuclear localization sequences (NLS) in its FERM domain^16^. However, the potential role of nuclear FAK and in particular whether FAK signaling can also impact on VEGFR expression or functions in the nucleus of ECs to promote angiogenesis remains to be determined.

Recent studies using EC-specific FAK conditional KO and kinase-defective (KD) mutant knockin mouse models demonstrated both the kinase-dependent and kinase-independent functions of FAK in embryonic angiogenesis^17–19^. The potential role of FAK in adult angiogenesis has also been examined by inducible EC-specific deletion of FAK, but with less conclusive results^20–22^. In one study, no apparent angiogenesis defect was detected using matrigel plug and aortic ring assays due to compensatory Pyk2 up-regulation^20^, although the mutant mice exhibited defective vascular permeability induced by VEGF^22^. In contrast, the other study showed decreased tumor angiogenesis and altered blood vessel density in sponge assays in the mutant mice^21^. Although the different methods and experimental conditions in the two studies may have contributed to the discrepancy, this discrepancy highlights the importance for further investigations to clarify the role of FAK in adult angiogenesis. Moreover, the underlying mechanisms, especially the downstream targets of FAK signaling in the regulation of EC function and angiogenesis in adult organisms, remain to be characterized.

Here, we have generated endothelial-specific tamoxifen-inducible FAK knockout mice and FAK kinase-defective (KD) knockin mice to determine the role and mechanisms of FAK and its kinase activity in the regulation of angiogenesis in adult mice. We identify a novel function of FAK to regulate VEGFR2 expression to promote EC proliferation and migration as well as angiogenesis in adult mice *in vivo*. FAK regulation of VEGFR2 requires both its nuclear localization and its kinase activity and appears to be mediated by the direct association of nuclear FAK in the polymerase II complex on VEGFR2 promoter.

## Results

### FAK and its kinase activity are required for angiogenesis in adult mice

**Figure 1:**
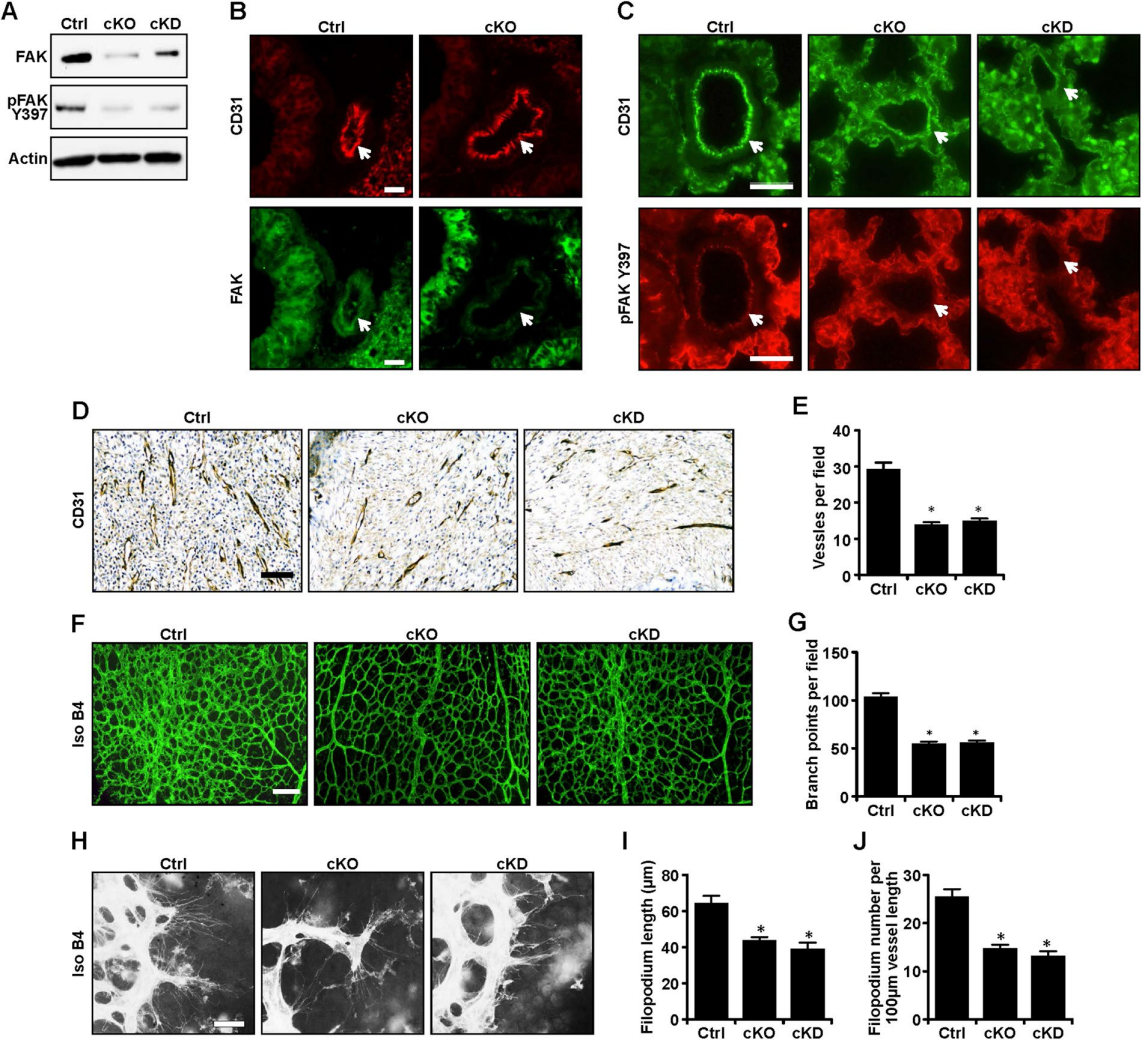
FAK ablation or kinase inactivation decreases postnatal angiogenesis in mice. (**A**) Lysates from primary lung ECs of control, cKO and cKD mice were analyzed by immunoblotting using various antibodies as indicated. (**B**,**C**) Representative images of immunofluorescence staining of lung sections of control, cKO and cKD mice by CD31, FAK and pFAK, as indicated. Arrows mark vessels. Scale bar = 25 μm. (**D**,**E**) Sections were prepared from granulation tissues of control, cKO and cKD mice at 7 days after wounding and analyzed by IHC for CD31. Representative images are shown in (**D**), and quantification of numbers of vessels per field is shown in (**E**). Scale bar = 50 μm. n = 5, mean ± SEM. *p < 0.05. (**F**,**G**) Representative images of whole-mount staining for isolectin B4 of retinas from control, cKO and cKD mice mice at P5 (**F**), and quantification of branch points per field (**G**). n = 5, mean ± SEM. *p < 0.05. Scale bar = 100 μm. (**H–J**) Representative images of filopodia from higher magnification of whole-mount staining for isolectin B4 of retinas from control, cKO and cKD mice at P5 (**H**), and quantification of average length (**I**) and number (**J**) of filopodia of tip cells. Scale bar = 25 μm. n = 5, mean ± SEM. *p < 0.05. Full-length western blots are presented in Supplementary Figure 1.

To investigate the role and mechanisms of FAK and its kinase activity in angiogenesis of adult mice, FAK^f/f^ mice^18^ were crossed with End-Scl-Cre-ER^T^ transgenic mice that express tamoxifen-activated Cre recombinase^23^ in ECs to produce FAK^f/f^; Scl-Cre mice, which were then crossed with FAK^f/kd^ mice^19^ to generate FAK^f/kd^; Scl-Cre mice. Tamoxifen administration induces activation of Cre in ECs, leading to EC-specific depletion of FAK in FAK^f/f^; Scl-Cre (designated as cKO) mice and the expression of only FAK KD allele in FAK^f/kd^; Scl-Cre (designated as cKD) mice. To validate the inducible deletion, primary ECs were isolated from the lungs of cKO, cKD or control (FAK^f/f^ mice treated with tamoxifen, Ctrl) mice, as described previously^24^. Western blotting analyses showed significantly reduced FAK levels in ECs from cKO compared to those from Ctrl and cKD mice (Fig. 1A). Consistent with the inactivation of FAK kinase activity of KD mutation, phospho-FAK Y397 levels were reduced in ECs from cKO mice as well as cKD mice compared to those from Ctrl mice. Moreover, double-labeling immunofluorescence using lung sections showed FAK expression in ECs (as marked by CD31 co-staining) of the Ctrl, but not cKO mice (Fig. 1B). Further, phospho-FAK Y397 staining was also significantly reduced in ECs of both cKO and cKD lung sections relative to ECs of Ctrl mice (Fig. 1C).

We then used the previously described wound healing angiogenesis assay^25^ to assess the effect of endothelial FAK deletion and inactivation on angiogenesis in cKO and cKD mice. As shown in Fig. 1D and E, examination of the neovascularization in granulation tissue by staining of CD31 showed decreased capillary densities in both cKO and cKD mice compared to Ctrl mice. We next employed the neonatal mouse retinal angiogenesis model to examine how FAK and its kinase activity affect neonatal angiogenesis. The retina is avascular at birth, and a superficial vascular plexus grows progressively from the center toward the periphery during week 1 after birth^26^. The newborn pups were injected peritoneally with tamoxifen (100 ug per day from postnatal day 1 (P1) to P3). Examination of the retinal vasculature at P5 by isolectin B4 staining showed significantly decreased vascular branching in cKO and cKD retinas compared to that of Ctrl mice (Fig. 1F and G). Examination of the samples under higher magnification showed a significant reduction in the number and lengths of filopodia in tip cells in cKO and cKD retinas compared to Ctrl (Fig. 1H–J). Together, these results suggest that FAK and its kinase activity play important roles in promoting postnatal angiogenesis.

### Decreased EC proliferation and migration upon FAK deletion or kinase inactivation

**Figure 2:**
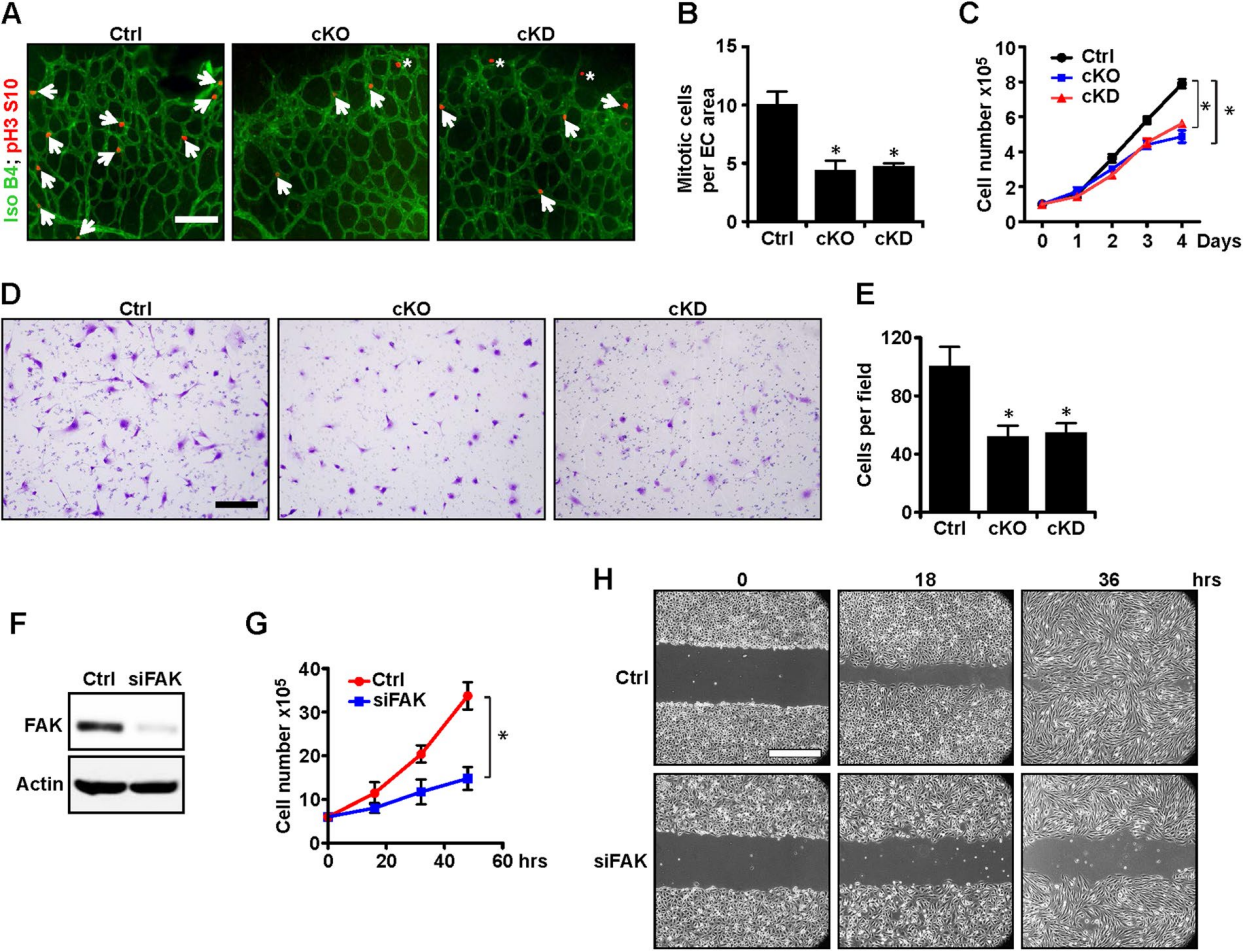
FAK and its kinase activity are required for VEGF-induced proliferation and migration. (**A**,**B**) Representative images of isolectin B4 (green) and pH3 (red) staining of whole-mount retinal vasculatures of control, cKO and cKD mice at P5 (**A**) and quantification of mitotic ECs (pH3 positive, IB4 positive, marked by arrows) per EC area. pH3 positive, IB4 negative cells are mitotic non-ECs, marked by asterisks. (**B**). Scale bar = 100 μm. n = 5, mean ± SEM. *p < 0.05. (**C**) Primary ECs were isolated from lungs of control, cKO and cKD mice, and cultured with VEGF for various days as indicated. n = 3, mean ± SEM. *p < 0.05. (**D**,**E**) Representative images of primary ECs from control, cKO and cKD mice that had migrated through membranes in response to VEGF in Transwell assays (**D**), and quantification of number of cells per field (**E**). Scale bar = 100 μm. n = 3, mean ± SEM. *p < 0.05. (**F**–**H**) MS1 cells were treated with siRNA for FAK or a control sequence, as indicated. Aliquots of lysates from the cells were analyzed by western blotting to show the efficiency of FAK knockdown (**F**). The cells were also analyzed for growth (**G**), n = 5, mean ± SEM. *p < 0.05.and migration using wound closure assay (**H**). Scale bar, 250 μm. Full-length western blots are presented in Supplementary Fig. 2.

To investigate the potential mechanisms of reduced angiogenesis in cKO and cKD mice, we examined EC proliferation in the sprouting vessels by staining for phosphorylated histone H3 (pH3) to identify mitotic cells (Fig. 2A, arrows). Quantitative analysis of pH3 labeling showed significant reductions in the number of mitotic cells per EC area in cKO and cKD retinas (Fig. 2B). Given the critical role of VEGF during neonatal retinal angiogenesis^27^, we examined proliferation of primary ECs isolated from lungs of Ctrl, cKO and cKD mice in response to VEGF. Similar to previous observations^18^, FAK-null ECs from cKO mice showed decreased proliferation compared to Ctrl ECs (Fig. 2C). Moreover, cKD ECs lacking FAK kinase activity also showed a comparable decrease, suggesting an essential role for FAK kinase function to promote VEGF-stimulated EC proliferation. We also examined the effects of FAK deletion and its kinase inactivation on VEGF-regulated cell migration in primary ECs. We found that ECs from cKO and cKD mice showed significantly decreased chemotaxis in response to VEGF relative to Ctrl ECs as measured by Boyden chamber assays (Fig. 2D and E). We further evaluated the role of FAK in ECs employing an established mouse EC line MS1 cells. A similar decrease in proliferation was observed in MS1 cells upon FAK knock down using siRNA (Fig. 2F and G). Likewise, FAK knockdown also reduced migration of MS1 cells as measured by wound closure assays (Fig. 2H). Together, these results demonstrate that ablation of FAK or its kinase activity inhibits VEGF-induced EC proliferation and migration, which may lead to the reduced angiogenesis observed in cKO and cKD mice.

### FAK and its kinase activity control VEGFR2 transcription in ECs

**Figure 3:**
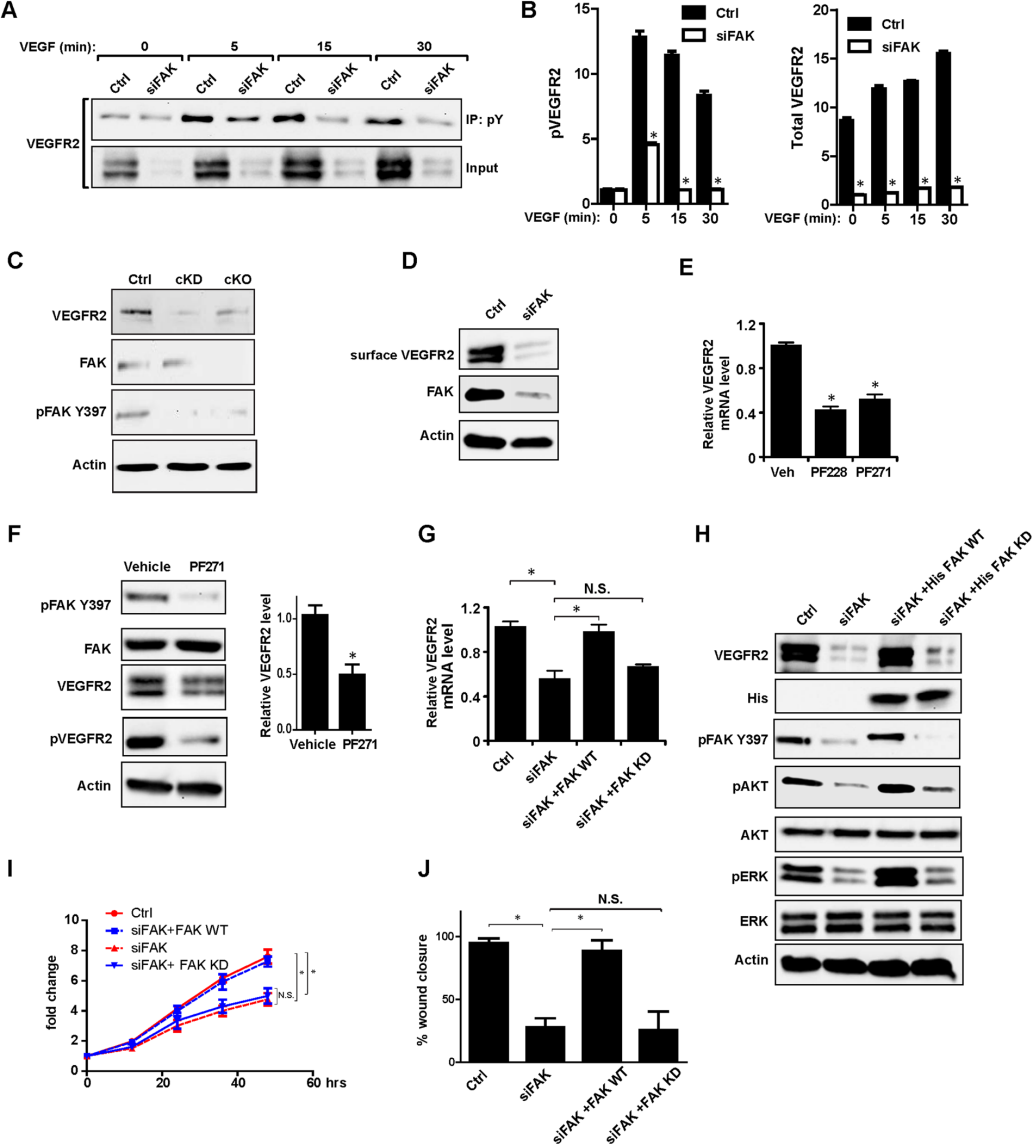
Regulation of VEGFR2 by FAK and its kinase activity in ECs. (**A**) Lysates from MS1 cells with or without FAK knockdown were immunoprecipitated with anti-phosphotyrosine antibody 4G10 and analyzed by immunoblotting using VEGFR2 antibody as indicated. The relative expression levels of pVEGFR2 and VEGFR2 are quantified (**B**). n = 3, mean ± SEM. *p < 0.05. (**C**) Lysates from primary lung ECs of control, cKO and cKD mice were analyzed by immunoblotting using various antibodies as indicated. (**D**) MS1 cells were transfected with FAK siRNA for FAK or a control siRNA. Surface VEGFR2 as labeled by biotin was pulled down by streptavidin-conjugated agarose beads, and checked using VEGFR2 antibody. Aliquots of the whole cell lysates were also analyzed directly by western blotting with antibodies as indicated. (**E**) MS1 cells were treated with FAK kinase inhibitors PF228 or PF271 as well as corresponding vehicle. mRNAs were prepared and analyzed by qRT-PCR for relative *VEGFR2* mRNA levels (normalized to *GAPDH* mRNA level, Vehicle treated cells as 1). n = 3, mean ± SEM. *p < 0.05. (**F**) MS1 cells were treated with FAK kinase inhibitors PF271 and vehicle. Lysates were analyzed by immunoblotting using various antibodies as indicated. To check the phosphorylation of VEGFR2, Lysates were immunoprecipitated with anti-phosphotyrosine antibody 4G10 and analyzed by immunoblotting using VEGFR2 antibody. The relative expression levels of VEGFR2 are quantified. n = 3, mean ± SEM. *p < 0.05. (**F**) MS1 cells were co-transfected with FAK siRNA and expression vectors encoding wild type or kinase-defective FAK, as indicated. mRNAs were prepared and analyzed by qRT-PCR for relative *VEGFR2* mRNA levels (normalized to *GAPDH* mRNA level, and Ctrl cells as 1). n = 3, mean ± SEM. *p < 0.05. N.S., not significant. (**H**) MS1 cells were co-transfected with FAK siRNA and expression vectors encoding wild type or kinase-defective FAK. Lysates were analyzed by immunoblotting using various antibodies as indicated. (**I**) Cell proliferation was monitored by analyzing the relative occupied area of cell images over time. n = 3, mean ± SEM. *p < 0.05. (**J**) Cell migration was monitored by analyzing the % wound closure area of cell images over time. n = 3, mean ± SEM.*p < 0.05. Full-length western blots are presented in Supplementary Figures 3–6.

To explore the roles of FAK and its kinase activity in the regulation of ECs by VEGF signaling, we first analyzed the activation of AKT and ERK1/2 upon VEGF stimulation, both well characterized downstream targets of VEGF receptors^28^. Consistent with previous findings, FAK knockdown in MS1 cells reduced both basal and VEGF-stimulated AKT and ERK activation (Data not shown), verifying that FAK is essential for VEGF signaling. We therefore examined the possibility that FAK may directly affect VEGFR2, the major mediator for VEGF signaling in ECs^28^ to impact on its downstream signaling to AKT and ERK by measuring its auto-phosphorylation. Compared to control siRNA treated cells, FAK knockdown significantly decreased the level of phosphorylated VEGFR2, and analysis of total VEGFR2 levels showed that this reduction was caused by the decreased expression of VEGFR2 in FAK knockdown cells (Fig. 3A and B). We also compared the expression level of VEGFR2 in primary ECs from control, cKD and cKO mice. We found that the expression level of VEGFR2 in primary ECs from cKD and cKO mice was much lower comparing to the control mice (Fig. 3C). Consistent with the decreased amount of VEGFR2, FAK knockdown also significantly decreased the level of surface VEGFR2 (Fig. 3D). Moreover, FAK knockdown by siRNA and inhibition of its kinase activity by PF-228 or PF271 significantly reduced VEGFR2 protein and mRNA levels (Fig. 3E and F), suggesting that FAK and its kinase activity regulated VEGFR2 expression at the transcriptional level. To further validate the role of FAK and its kinase activity in the regulation of VEGFR2 expression, MS1 cells with FAK knockdown were transfected with expression vectors encoding wild type FAK or kinase-defective FAK mutant. We found that re-expression of FAK, but not kinase-defective FAK mutant, rescued the decreased expression of VEGFR2 in FAK-depleted MS1 cells, VEGF signaling as measured by phosphorylation of AKT and ERK as well as the decreased proliferation and migration of these cells (Fig. 3G–J). Collectively, these results indicate that FAK and its kinase activity promote VEGFR2 expression at transcriptional level in ECs.

### VEGF and FN-induced FAK translocation into the nucleus in ECs

**Figure 4:**
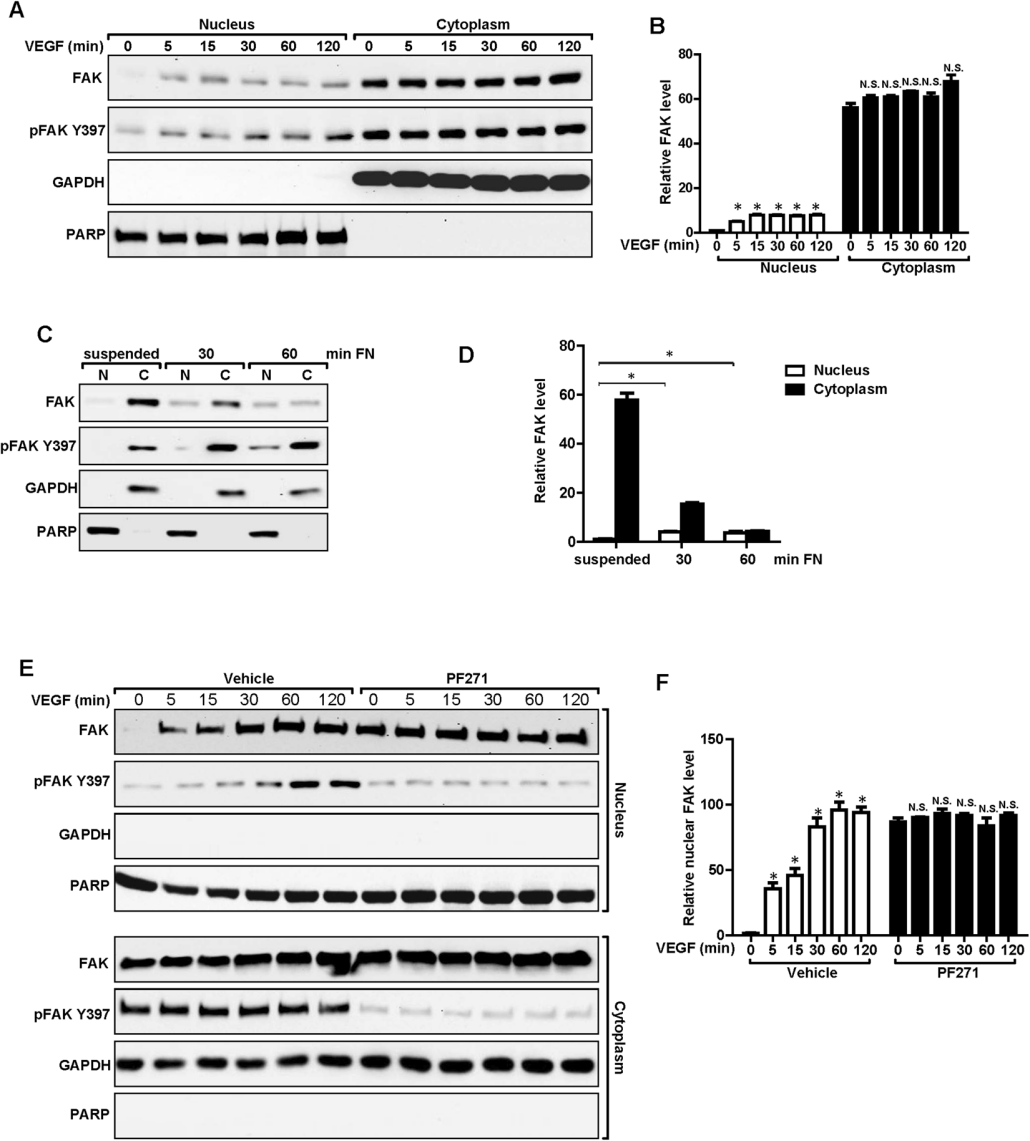
Nuclear translocation of FAK in ECs. (**A**,**B**) MS1 cells were stimulated with 30 ng/ml VEGF for indicated times. They were lysed and fractionated to cytoplasmic and nuclear portions, and analyzed by immunoblotting using indicated antibodies. GAPDH was used as a cytoplasmic marker and RARP as a nuclear marker (**A**). The relative levels of FAK are quantified (**B**). n = 3, mean ± SEM. *p < 0.05. N.S., not significant. (**C**,**D**) MS1 cells were held in suspension for 30 min, and re-plated for 30 or 60 min on dishes coated with 10 μg/ml fibronectin. They were then analyzed as in A (**C**). The relative levels of FAK are quantified (**D**). n = 3, mean ± SEM. *p < 0.05. (**E**,**F**) MS1 cells were treated with FAK kinase inhibitor PF-271 or vehicle for 12 hours followed by VEGF stimulation for indicated times. They were then analyzed as in C. The relative levels of nuclear FAK are quantified (**F**). n = 3, mean ± SEM. *p < 0.05. Full-length western blots are presented in Supplementary Figures 7–9.

Recent studies suggested that FAK can translocate into the nucleus and take part in the regulation of Ccl5 transcription in squamous cell carcinoma cells or p53 degradation in fibroblasts^16,29^. To determine whether regulation of VEGFR2 transcription is also through nuclear FAK, we first examined possible FAK translocation into the nucleus in ECs upon VEGF stimulation. Lysates were prepared from the cytoplasmic and nuclear fractions of MS1 cells that had been treated with VEGF for various times and then subjected for Western blotting analysis. As expected, the majority of FAK was present in the cytoplasm before VEGF addition and at different times of VEGF stimulation. However, a small amount of FAK can be detected in the nuclear fraction at basal level, and its presence in the nucleus was significantly increased upon VEGF stimulation at 5 min and thereafter (Fig. 4A and B). We also evaluated FAK nuclear translocation in response to fibronectin-mediated cell adhesion. We found that while minimal amount of FAK was present in the nucleus of suspended MS1 cells, significantly increased fraction of FAK was found in the nucleus upon cell adhesion to fibronectin (Fig. 4C and D). Because both VEGF and fibronectin are known to stimulate FAK phosphorylation^12,14^, which were also observed in MS1 cells (Fig. 4A and C), we next examined whether FAK kinase activity and its consequent auto-phosphorylation is required for its efficient translocation into the nucleus. Treatment of MS1 cells with FAK inhibitor PF-271 did not reduce, but surprisingly increased, nuclear localization of FAK (Fig. 4E and F). These data indicated that the nuclear translocation of FAK is independent of its kinase activity or auto-phosphorylation at Y397. These data indicated FAK can translocate into the nucleus independent of its kinase activity or auto-phosphorylation at Y397, and that FAK inhibitor treatment could trap it in the nucleus.

### Nuclear FAK regulates the transcription of VEGFR2

**Figure 5:**
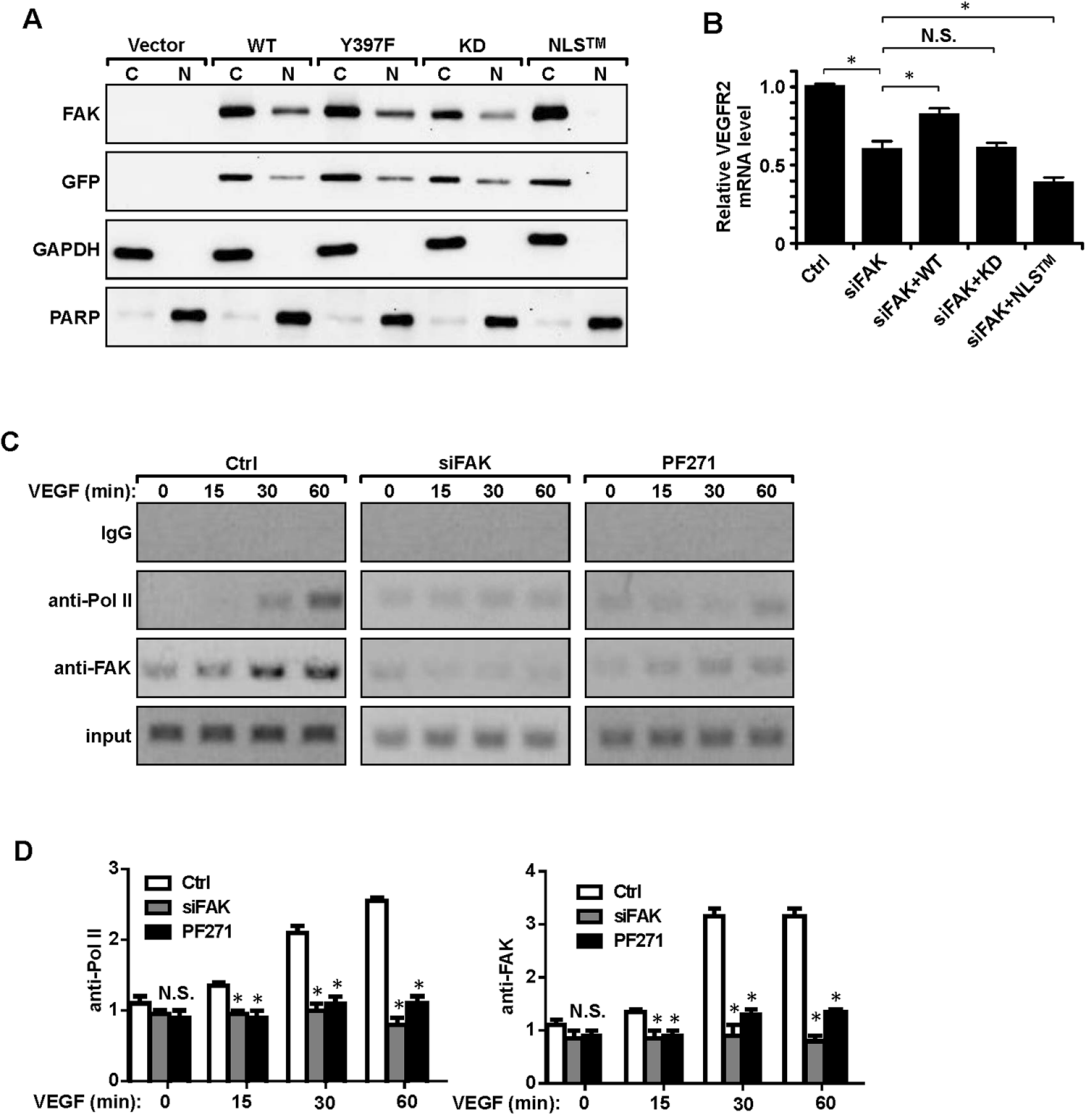
Nuclear FAK regulates the transcription of VEGFR2. (**A**) FAK^−/−^ MEFs were transfected with different GFP-FAK mutant constructs, as indicated. They were then fractionated and analyzed as in Fig. 4 using various antibodies as indicated. (**B**) MS1 cells were co- transfected with FAK siRNA and indicated FAK constructs. mRNAs were then prepared and analyzed by qRT-PCR for relative *VEGFR2* mRNA levels (normalized to *GAPDH* mRNA level, and Ctrl cells as 1). n = 3, mean ± SEM. *p < 0.05. (**C**,**D**) MS1 cells were treated with siRNA for FAK for 48 hours or FAK inhibitor PF-271 for 12 hours, followed by VEGF stimulation for indicated times. ChIP assays were then performed with antibodies directed against FAK, RNA polymerase II or IgG as a control. The precipitated DNA was analyzed with the indicated primers for VEGFR2 promoter. Input represents 1% of the genomic DNA used. (**D**) The quantification for FAK and Pol ii. n = 3, mean ± SEM. *p < 0.05. N.S., not significant. Full-length western blots and gels are presented in Supplementary Figures 10 and 11.

Although FAK signaling may promote VEGFR2 expression at the transcriptional level by various direct or indirect mechanisms given its impact on many intracellular signaling pathways, the nuclear localization of FAK in ECs raised the interesting possibility that FAK promoted VEGFR2 transcription directly in the nucleus. To examine this possibility, we generated a FAK mutant with all three clusters of R/K nuclear localization signals mutated to As (i.e. R177/178 A, K190/191 A, K216/218 A, designated as NLS^TM^ mutant) based on previous studies in other cells^16^. We first examined the NLS^TM^ mutant as well as wild type FAK and several other mutants in FAK^−/−^ MEFs and verified that it indeed blocked FAK translocation to the nucleus (Fig. 5A). In contrast, wild-type as well as both Y397F and kinase-defective FAK mutants showed comparable levels of nuclear localization, which is consistent with our observation that inhibition of FAK kinase activity and consequent Y397 auto-phosphorylation did not reduce FAK localization to the nucleus in ECs. We then re-expressed the NLS^TM^ mutant as well as other constructs in MS1 cells with FAK knockdown and examined its effect on VEGFR2 transcription (Fig. 5B). As observed earlier, wild-type, but not kinase-defective FAK, rescued the decreased levels of VEGFR2 mRNA in these cells. Interestingly, the NLS^TM^ mutant could not restore it, but indeed further decreased the level of VEGFR2 (compare lane siFAK + NLS^TM^ vs siFAK alone). This might be due to an incomplete knockdown of FAK (i.e. there is still some level of FAK in siFAK cells) and a possible dominant negative effect of the NLS mutant (i.e. blocking the activity of residual FAK in siFAK cells). Together, these results suggest that nuclear localization of FAK is required for its promotion of VEGFR2 transcription.

To further explore whether FAK may participate directly in the RNA polymerase II-mediated transcription of VEGFR2, we examined the potential interaction of FAK with the VEGFR2 promoter region using chromatin immunoprecipitation (ChIP) assays. DNAs immunoprecipitated by anti-FAK, anti-RNA polymerase II or IgG as a control were analyzed by PCR using primers for the VEGFR2 promoter. VEGF stimulation increased the presence of RNA polymerase II in the VEGFR2 promoter (Fig. 5C and D). Interestingly, we also detected increasing association of FAK with the VEGFR2 promoter, suggesting a direct participation of nuclear FAK to enhance VEGFR2 transcription. Treatment of the cells with siRNA to knockdown FAK or PF271 to inhibit its activity abolished the presence of both RNA polymerase II and FAK in the complex, which is consistent with their inhibition of VEGFR2 expression as measured by its mRNA levels (see Fig. 3D and E). Together, these results suggest that FAK regulates the transcription of VEGFR2 directly through its nuclear translocation and in a kinase activity-dependent manner.

## Discussion

By generation and analysis of a KD FAK knockin mutant mice, we showed recently that FAK regulated vascular development through both its kinase-independent and -dependent functions in the control of EC survival and their barrier roles, respectively, during embryogenesis^19^. Here, we investigated the roles of these FAK functions in adult ECs and revealed that both FAK ablation and the loss of its kinase activity led to similar defects in the reduced expression of VEGFR2, decreased EC proliferation and migration, and deficient angiogenesis, suggesting that FAK acts in adult ECs primarily through its kinase functions. These results suggest interesting differences in the requirement for the scaffolding function of FAK in embryonic and adult ECs. Recent studies by us and others also showed that FAK deletion triggered increased vascular permeability in the embryos^19^, but decreased it in adult mice^22^. The differential requirement for FAK and its kinase and non-kinase functions in embryonic and adult ECs could relate to their rapidly proliferative nature during embryogenesis while being relatively quiescent in adult mice, but further investigation will be required to understand the mechanisms involved.

Our results lend further support to the notion that FAK is required for angiogenesis, despite a previous report on the lack of angiogenesis defects after FAK deletion in ECs^20^. A compensatory up-regulation of Pyk2 (proline-rich tyrosine kinase 2), a FAK-related kinase, was found in the previous study^20^. However, we did not find any increased expression of Pyk2 in either cKO or cKD mice in this study (data not shown), which could explain the apparent difference in our observation and those of Weis *et al*.^20^. Another previous study showed that FAK deletion in ECs *in vitro* decreased VEGF-stimulated Akt phosphorylation and reduced their proliferation and survival, which could contribute to the decreased angiogenesis *in vivo* in this study^21^. Our studies provide further mechanistic insights into the regulation of angiogenesis by revealing a direct role for nuclear FAK in transcriptional regulation of VEGFR2, a central mediator of EC proliferation and migration in angiogenesis. We showed that, in addition to functioning downstream of VEGF signaling^13,14^, FAK can translocate into the nucleus to stimulate VEGFR2 expression directly to promote angiogenesis. These results are consistent with several recent studies showing a nuclear function of FAK in the regulation of Mdm2-dependent p53 degradation^16^, heterochromatin remodeling^15^, and chemokine transcription^29^ in various other cell types.

Our data showed that both FAK kinase activity and its nuclear translocation are required for promoting VEGFR2 expression to stimulate angiogenesis. However, FAK auto-phosphorylation and its nuclear localization appear to be two independent events, although both are induced by VEGF. Interestingly, we found increased FAK in the nucleus upon inhibition of its phosphorylation, which is consistent with a recent report that non-phosphorylated FAK was preferentially localized in the nucleus of MEFs^30^. Future studies are needed to determine how VEGF stimulates FAK nuclear translocation through mechanisms independent of inducing its phosphorylation. Likewise, it remains to be determined as to how FAK kinase activity promotes its transcription activity for VEGFR2 without promoting (or even inhibiting) its nuclear localization.

## Methods

### Mice

*FAK*^*f/f*^, *FAK*^*f/kd*^ and End-Scl-Cre-ER^T^ mice were described previously^18,19,23^. Male and female mice with more than 98% C57BL/6 backgrounds were used. Age- and littermate-matched control and mutant mice were randomly collected by genotype. Mice were housed and handled according to local, state, and federal regulations. All experimental procedures were carried out according to protocols approved by the Institutional Animal Care and Use Committees at the University of Cincinnati. All experiments were performed in accordance with relevant local, state and federal guidelines and regulations. To induce Cre activity in newborn mice, three consecutive IP tamoxifen injections (10 µl, 10 mg/ml in corn oil) (Sigma) were given on postnatal day P1, P2, and P3. To induce Cre activity in adult mice, three consecutive IP tamoxifen injections (100 µl, 10 mg/ml in corn oil) (Sigma) were given about 2 months after birth. Control *FAK*^*f/f*^ mice received the same tamoxifen treatment.

### ECs isolation and culture

Mouse EC isolation and culture was performed as published previously (Zhao *et al*., 2010). Briefly, lungs were harvested, minced, and digested with 0.2% type I collagenase (Worthington Biomedical) at 37 °C for 1 hr. The digested tissue was mechanically dissociated using vigorous flushing through a metal cannula, passed through a 70-μm filter (BD), and centrifuged at 400 g for 5 min at 4 °C. The cells were re-suspended in DMEM then incubated with rat anti–murine CD31 (MEC 13.3, BD) coated magnetic beads (M-450; sheep anti–rat IgG Dynabeads, Invitrogen) for 10 min at 4 °C. The beads were washed several times then re-suspended in DMEM containing 20% FBS, supplemented with 100 µg/ml porcine heparin, 25 mM HEPES, 100 µg/ml endothelial mitogen (Biomedical Technologies, Inc), nonessential amino acids, sodium pyruvate, L-glutamine, and antibiotics, at standard concentrations, and cultured in 0.1% gelatin (Sigma) coated 100-mm tissue culture dishes at 37 °C in 5% CO_2_. The endothelial identity of the cells was confirmed by staining with CD31 (1:50, Dianova DIA310). The purity of ECs is estimated as 99% or higher.

### Histology and immunostaining

Mouse tissues were kept in 10% formalin neutral buffer (Sigma) at 4 °C. The specimens were then processed, embedded in paraffin, and sectioned at 5 μm. For immunostaining, heat-induced antigen retrieval was done using a pressure cooker (Retriever 2000, PickCell Laboratories). Sections were incubated with primary antibodies at 4 °C overnight. For immunofluorescence, primary antibodies were detected by DyLight 488 or Texas Red-conjugated secondary antibodies (1:200, Jackson ImmunoResearch Laboratories, Inc). Primary antibodies used were phospho-histone H3 S10 antibody (1:100, Cell Signaling 9701), VEGFR2 (1:200, Cell Signaling 2479), CD31 (1:50, Dianova DIA310), CD31 (1:50, Dako m0823), Quantification used integral optical density by Image-Pro Plus software. For immunohistochemistry, the samples were incubated with biotinylated goat anti-rabbit IgG followed by streptavidin-HRP (ImmunoCruz Staining System, Santa Cruz, CA), using DAB as a substrate.

### Wound healing assay

Mice were anesthetized with ketamine/xylazine (100 mg/kg, 10 mg/kg) intraperitoneally for the surgical procedure, which was performed under aseptic conditions. Two full-thickness wounds were made on the backs of mice using a 2-mm skin punch. All mice are age matched. And the wound were made in exactly the same position in the backs of each animal. Seven days later, mice were euthanized and wounds were excised and fixed in 10% buffered formalin and stored. The sections through the wound were done in the same orientation between mice. They were then examined by immunohistochemistry using affinity purified polyclonal rabbit anti-CD31 as outlined above. The number of capillaries was counted in 10 random high (40×) magnification fields.

### Retinal whole mount staining

Eyes were fixed in 4% PFA at 4 °C for 2 hr and washed in PBS. Retinas were dissected, permeabilized and blocked in PBS containing 1% BSA and 0.5% Triton at 4 °C overnight, washed in PBLec (1% Triton X-100, 1 mM CaCl_2_, 1 mM MgCl_2_, and 0.1 mM MnCl_2_ in PBS), and incubated overnight in PBLec containing biotinylated isolectin B4 (1:25, Vector Labs). After three 20-minute washes in PBS, samples were incubated with FITC-streptavidin (1:100, Vector Labs) in PBS containing 0.5% BSA and 0.25% Triton X-100 at room temperature for 2 hr. Retinas were either flat mounted using Vectashield mounting medium (Vector Labs) or processed for labeling of mitotic cells using phospho-histone H3 S10 antibody (1:100, Cell Signaling 9701). The images have been taken in the same region of the retinas. The branching points, EC area, and mitotic cells per fields were quantified from multiple experiments as described previously (Gerhardt *et al*., 2003).

### Cell proliferation and migration assays

Primary ECs were isolated from lungs of control, cKO and cKD mice, and cultured in growth medium with VEGF (30 ng/ml VEGF). MS1 cells were purchased from ATCC and maintained in DMEM with 10% FBS. They were cultured in growth medium with VEGF (30 ng/ml VEGF). Cell proliferation for both cells was then measured by IncuCyte Live Cell Analysis System according to manufacturer’s instructions. Boyden chamber assay was performed to measure migration for primary ECs using 8-μm pore polycarbonate membranes as described previously^31^. Wound closure assay was performed to measure migration for MS1 cells as described previously^32^.

### Immunoblotting

Lysates were prepared from primary ECs or MS1 cells and analyzed by immunoblot. Antibodies used were phosphor-FAK (1:2000, Invitrogen 700255), FAK (1:2000, Cell Signaling 3285 S), phospho-AKT S473 (1:2000, Cell Signaling 4060), AKT (1:2000, Cell Signaling 9272), phosphor-ERK (1:2000, Cell Signaling 9101), PARP (1:2000, Cell Signaling 9532), betaActin(1:5000, Sigma A5441), VEGFR2 (1:1000, Cell signaling 2479), anti-phosphotyrosine (1:1000, Millipore 4G10). HRP-conjugated secondary antibodies (1:5000) were from Jackson ImmunoResearch Laboratories, Inc.

### siRNAs and transfection

The siRNAs against FAK were from Invitrogen (cat# 4390824, s11485). Cells were transfected with the indicated siRNA using Lipofectamine 2000 (Invitrogen) and used for experiments after 48 hr.

### Real-time PCR

Total RNA was isolated with GeneJET RNA purification kit (Thermo Scientific #K0731) according to the manufacturer’s instructions. Reverse transcription of purified RNA was performed using The iScript cDNA synthesis kit (BioRad 170-8891) with random primers. The quantification of gene transcripts was performed by real-time PCR using SYBR green I dye (Invitrogen). Expression values were normalized to control beta-Actin. The primers used are listed as follows: beta-Actin, 5′-AAAGACCTGTACGCCAACAC-3′ and 5′-GTCATACTCCTGCTTGCTGAT-3′. VEGFR2, 5′-GAAACAGGTGAGGTAGGCAG-3′ and 5′-ACCCTCGTTTTCAGAGTTGG-3′.

### ChIP assay

The ChIP assay kit (Millipore) was used according to the manufacturer’s instructions with some variations. Formaldehyde cross-linking was performed at room temperature for 10 min before glycine was added to a final concentration of 125 mM for 5 min. The cells were rapidly collected and lysed in SDS lysis buffer. Suspended chromatin was sheared by sonication to a mean size of 200–1,000 bp, centrifuged to pellet debris, and diluted 10 times with dilution buffer. Extracts were precleared for 2 h with salmon sperm DNA and BSA-saturated protein A/G beads. Immuno- precipitations were performed at 4 °C overnight using antibodies as indicated with IgG as a negative control. Immune complexes were collected and washed sequentially with Tris-SDS-EDTA buffer followed by two washes with Tris-EDTA buffer. Immune complexes were then extracted with elution buffer and DNA: protein complexes were disrupted by heating at 65 °C overnight. After proteinase K digestion for 1 h, DNA was extracted with provided column. About one twentieth of precipitated DNA was used as template in each PCR reaction. The following promoter-specific primers were used: VEGFR2, sense (CAGGACTGAAAGCCCAGACT) and antisense (CAGGCACAGACTCCTTCTCC); mouse GAPDH, sense (CACCATCCGGGTTCCTATAA) and antisense (AATCTCCACTTTGCCACTGC).

### Statistical analysis

Statistical significance for multivariable experiments was evaluated by ANOVA. p < 0.05 was considered to be statistically significant. Analyses used GraphPad Prism.

## Electronic supplementary material

Supplemental figure and legends
