# Supplementary material for: Nuclear FAK and its kinase activity regulate VEGFR2 transcription in angiogenesis of adult mice
Source: Sci Rep. 2018 Feb 7;8:2550. doi: 10.1038/s41598-018-20930-z (PMC5803223; doi:10.1038/s41598-018-20930-z)
Supplement: Supplementary file 1 — Supplemental figure and legends [file 41598_2018_20930_MOESM1_ESM.pdf]

Nuclear FAK and its kinase activity regulate VEGFR2 transcription in angiogenesis of adult mice

Shaogang Sun, Hsin-Jung Wu and Jun-Lin Guan

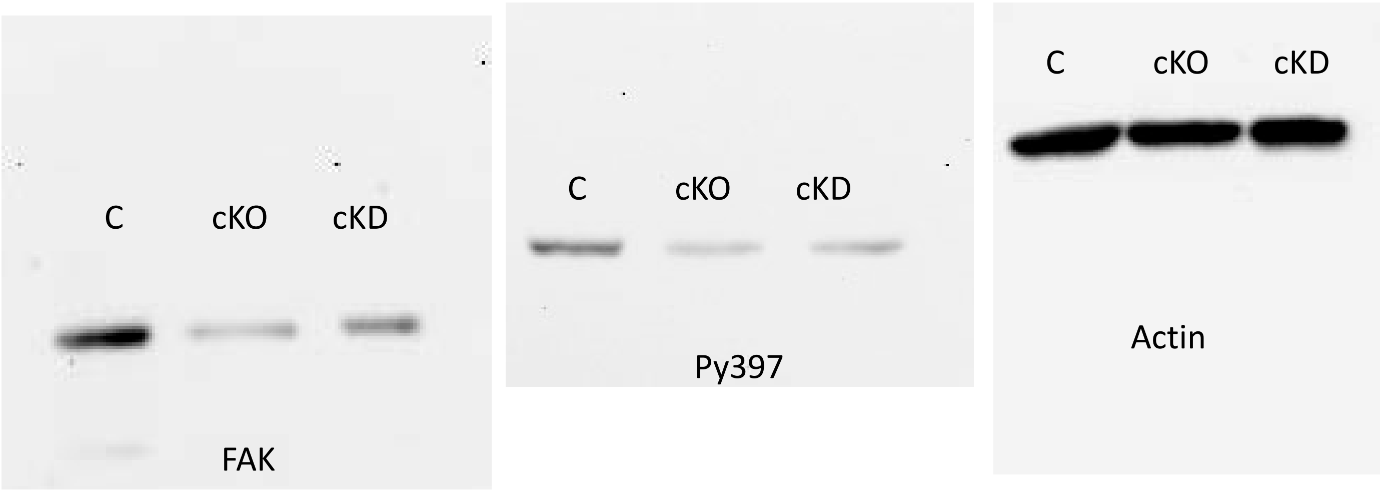

Supplement figure 1

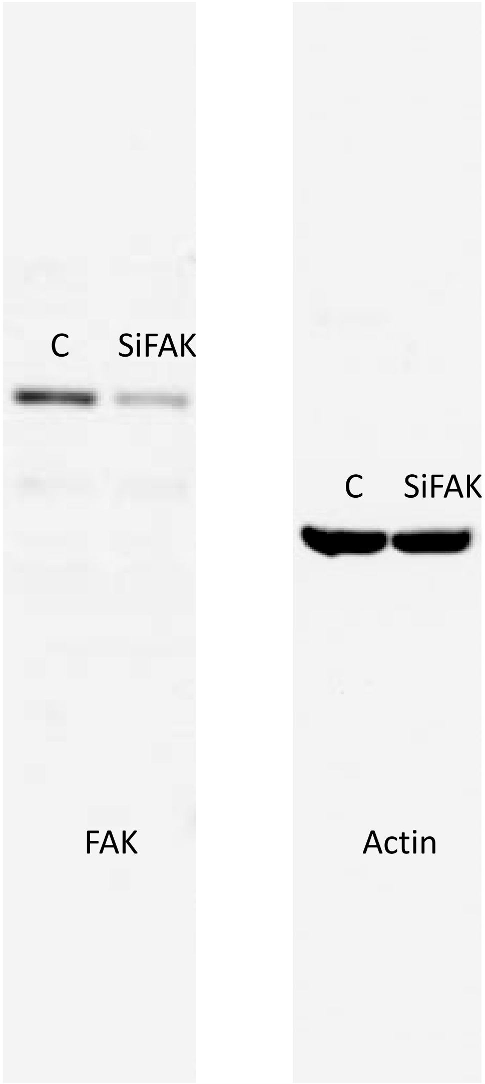

Supplement fig 2

A

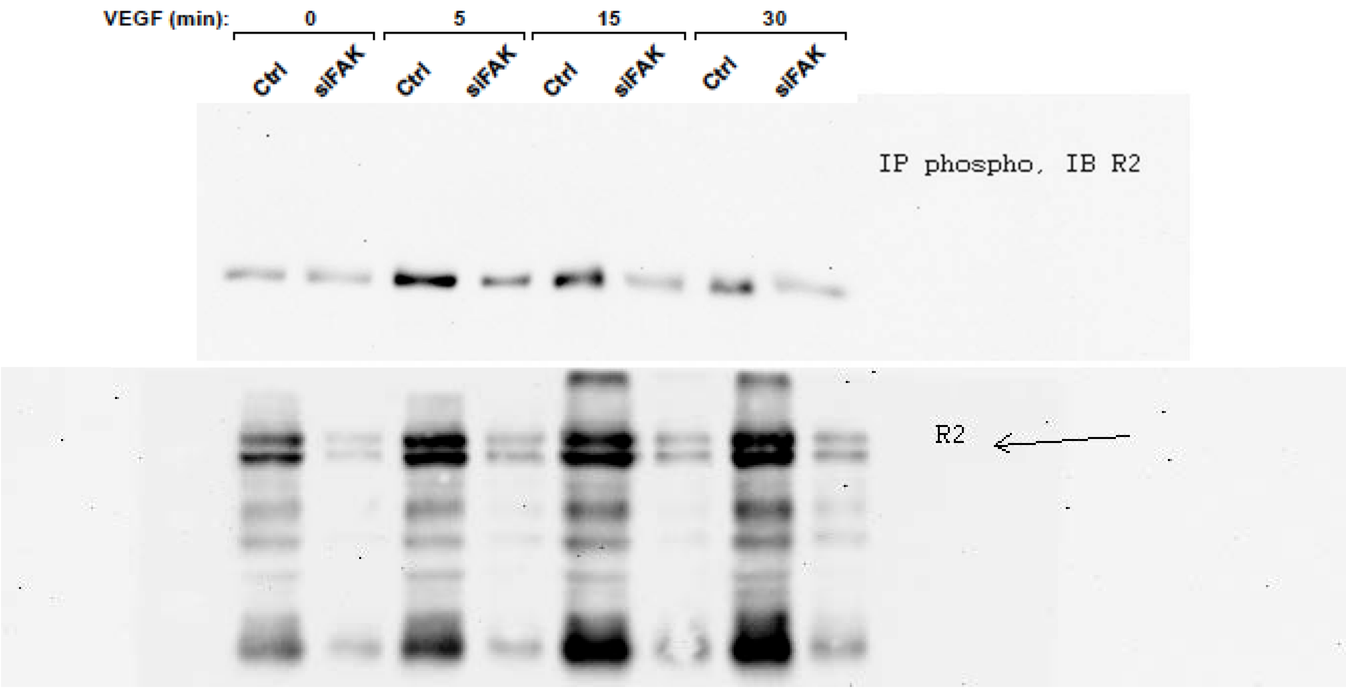

Supplement figure 3

C

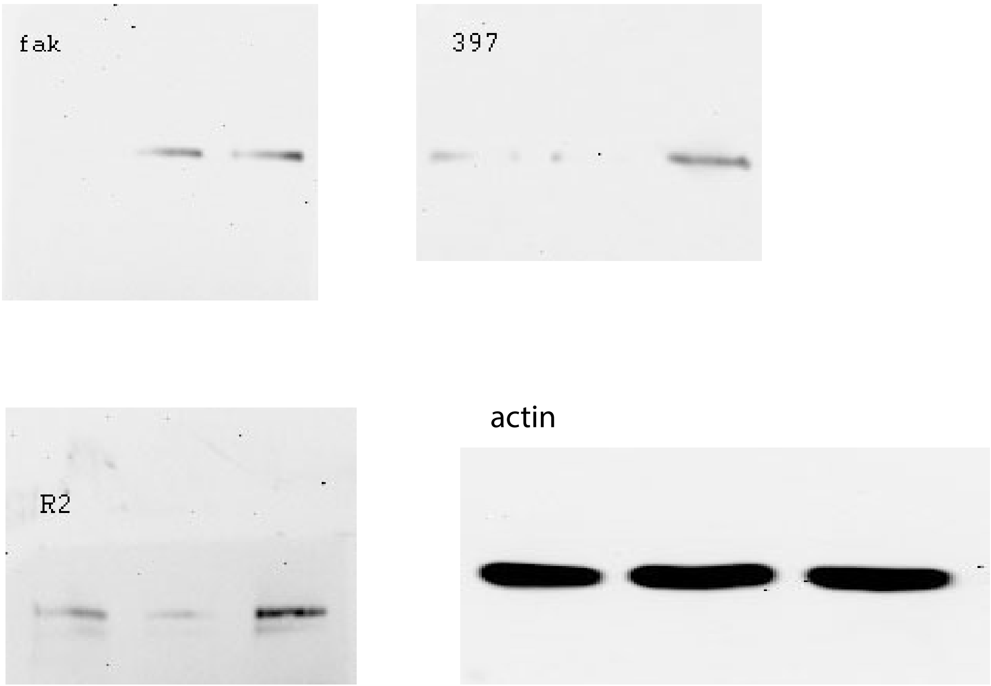

D

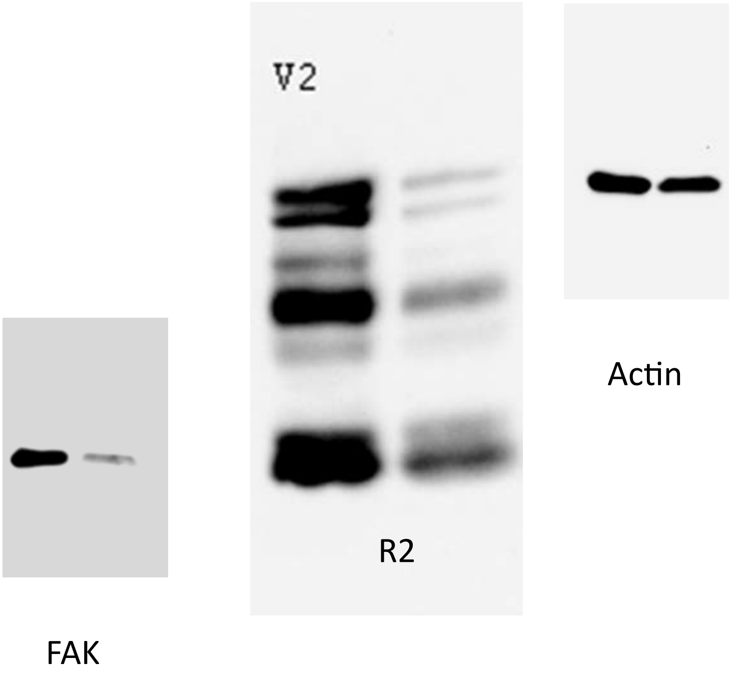

Supplement figure 4

F

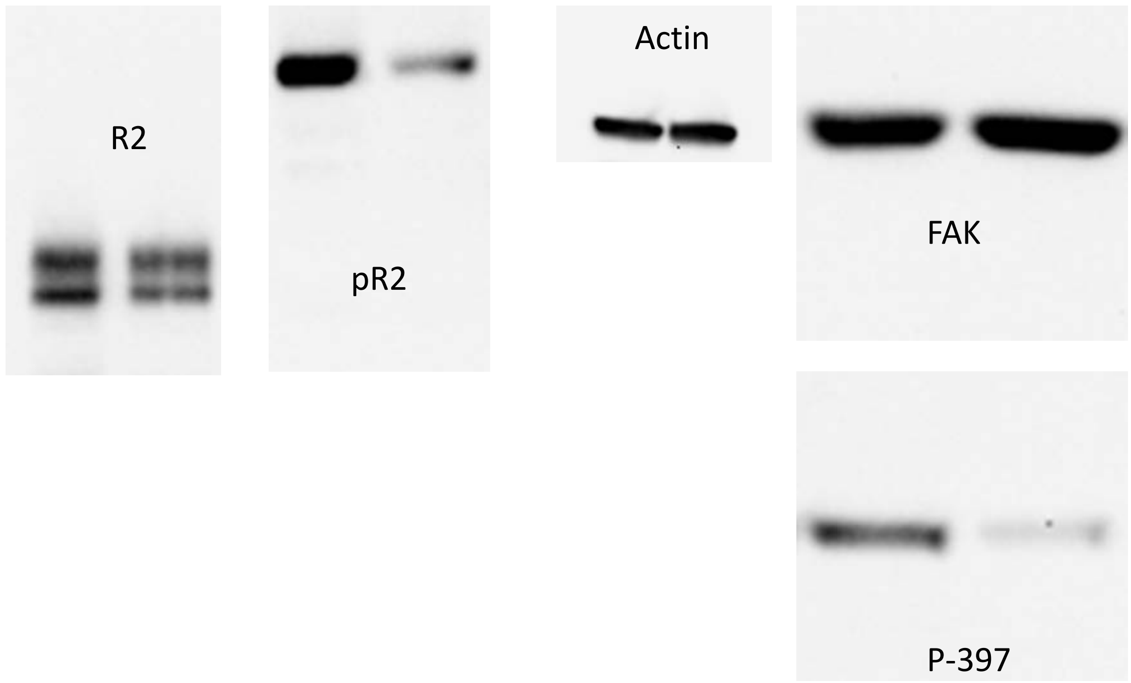

Supplement figure 5

H

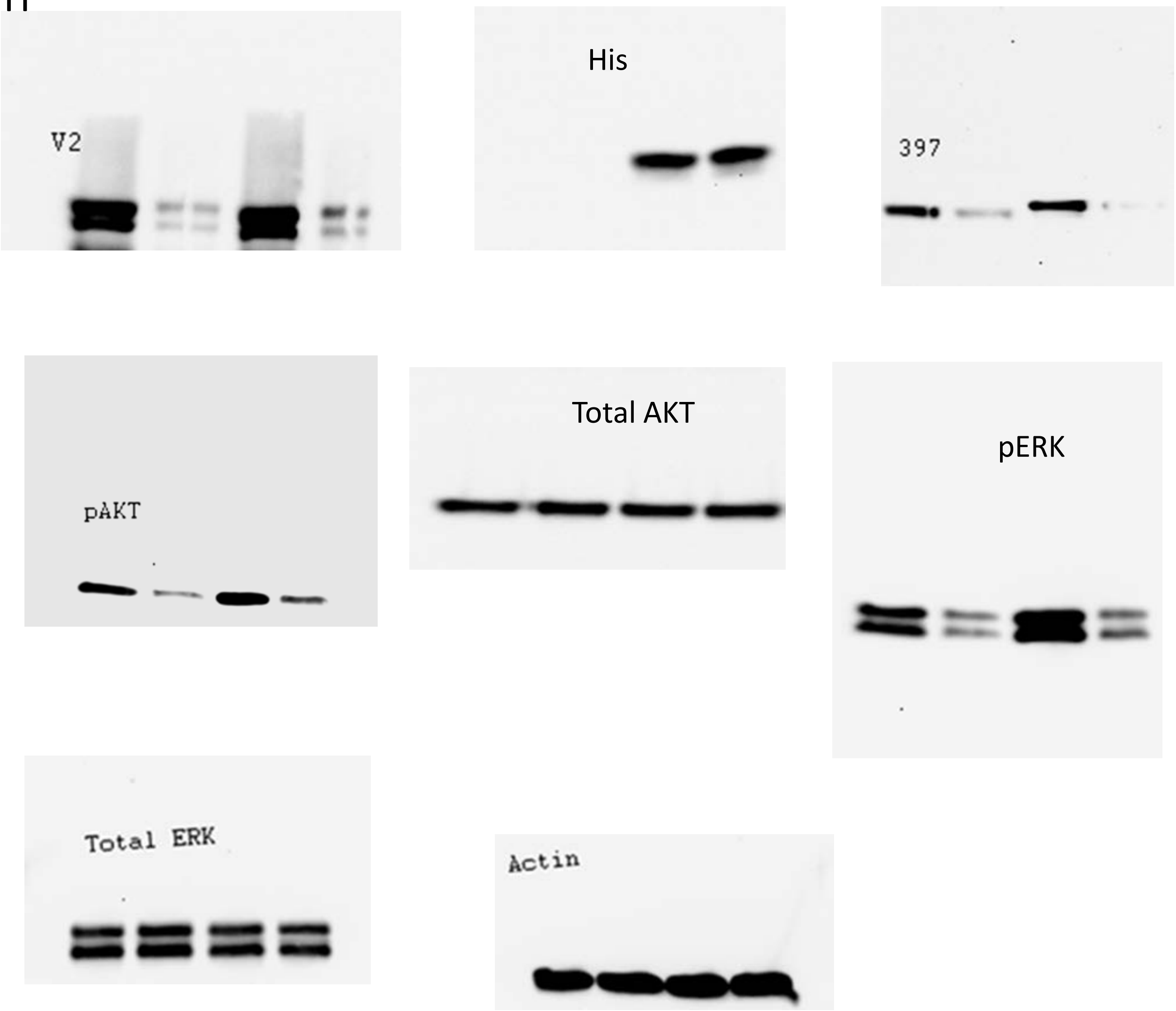

Supplement figure 6

4A

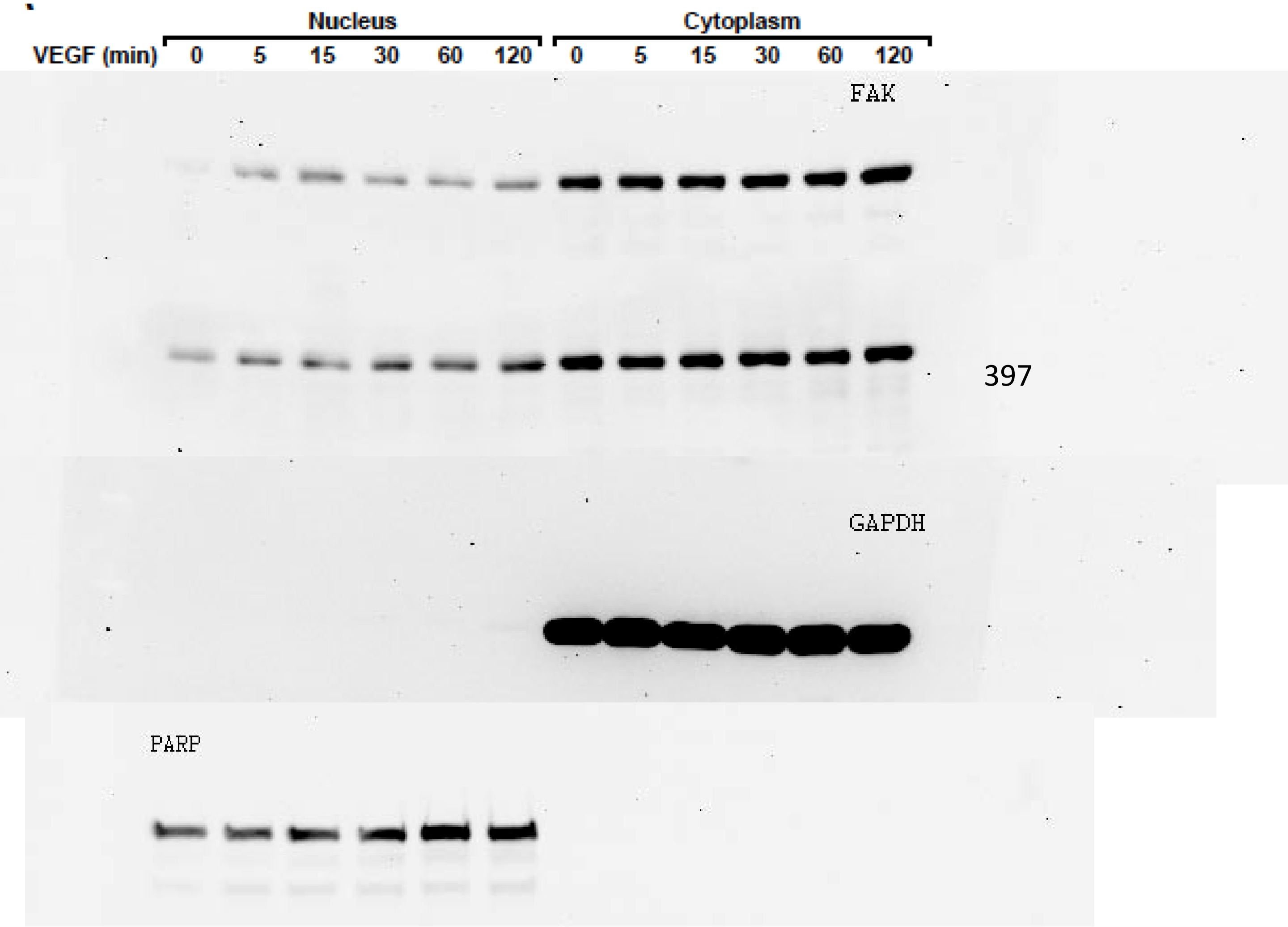

Supplement figure 7

4C

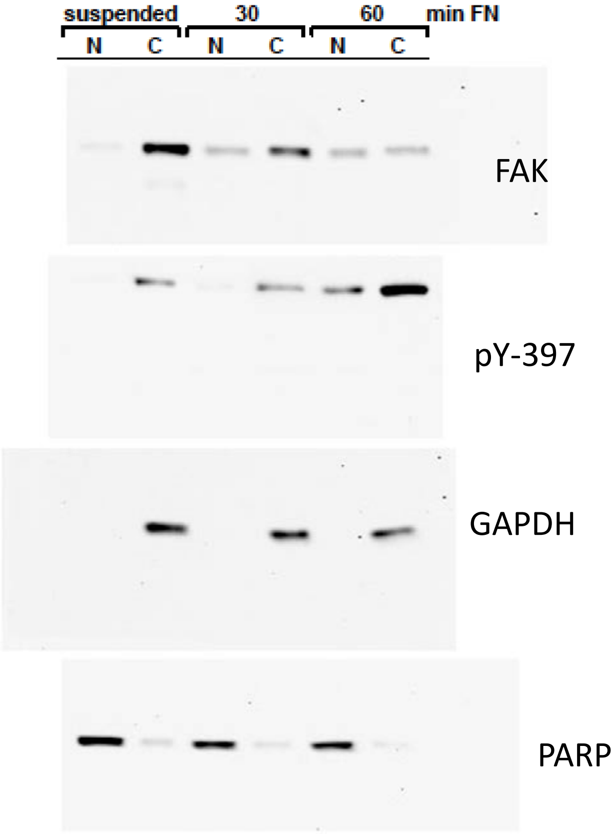

Supplement figure 8

4E

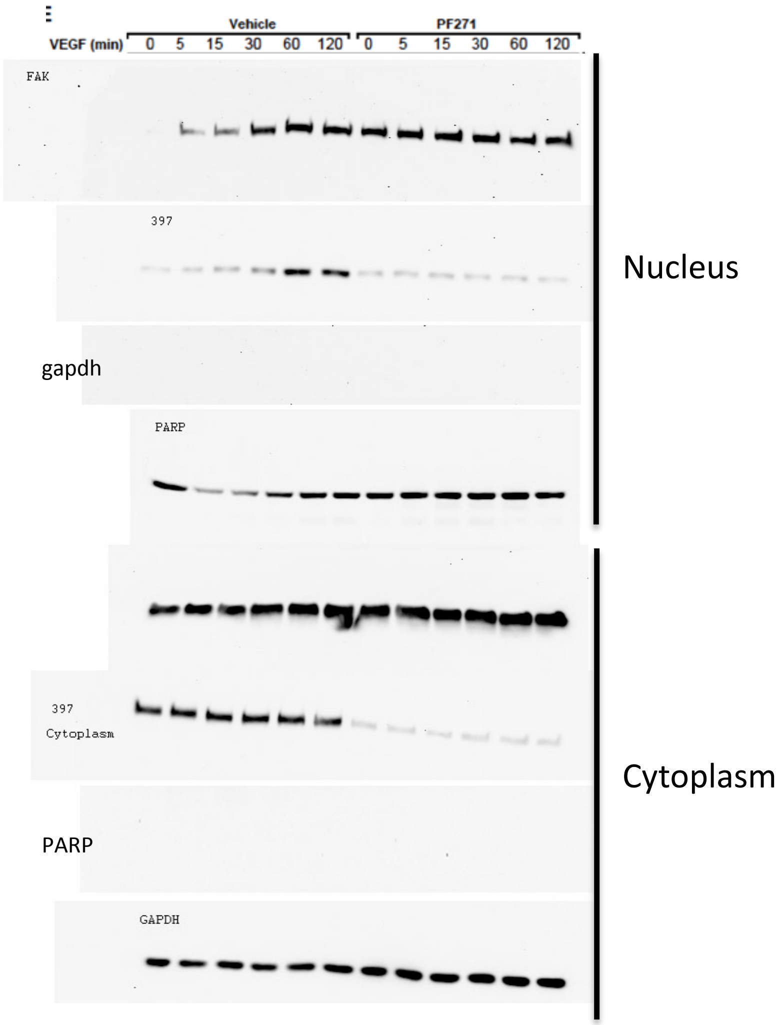

Supplement figure 9

5A

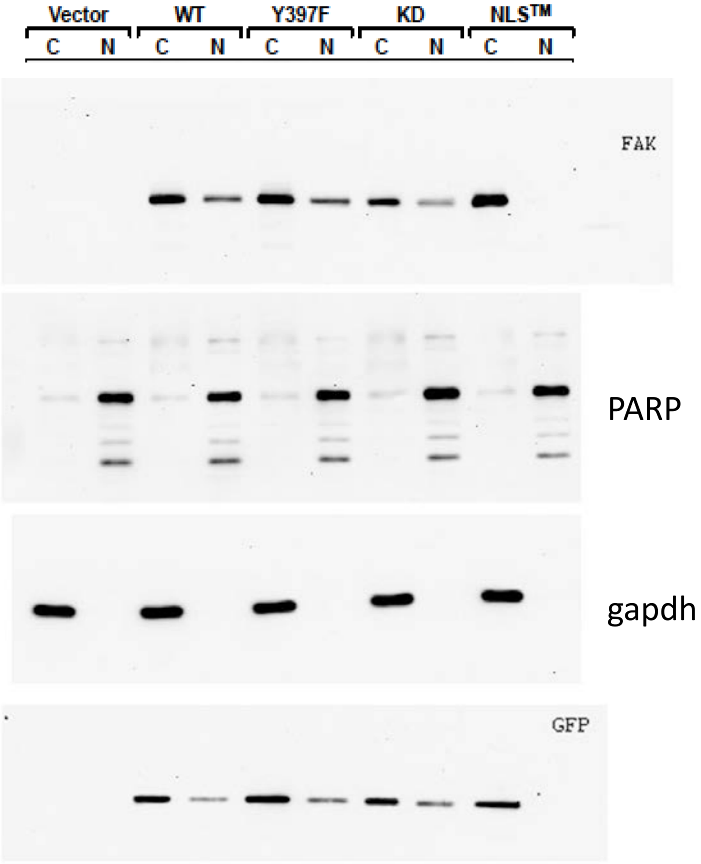

5C

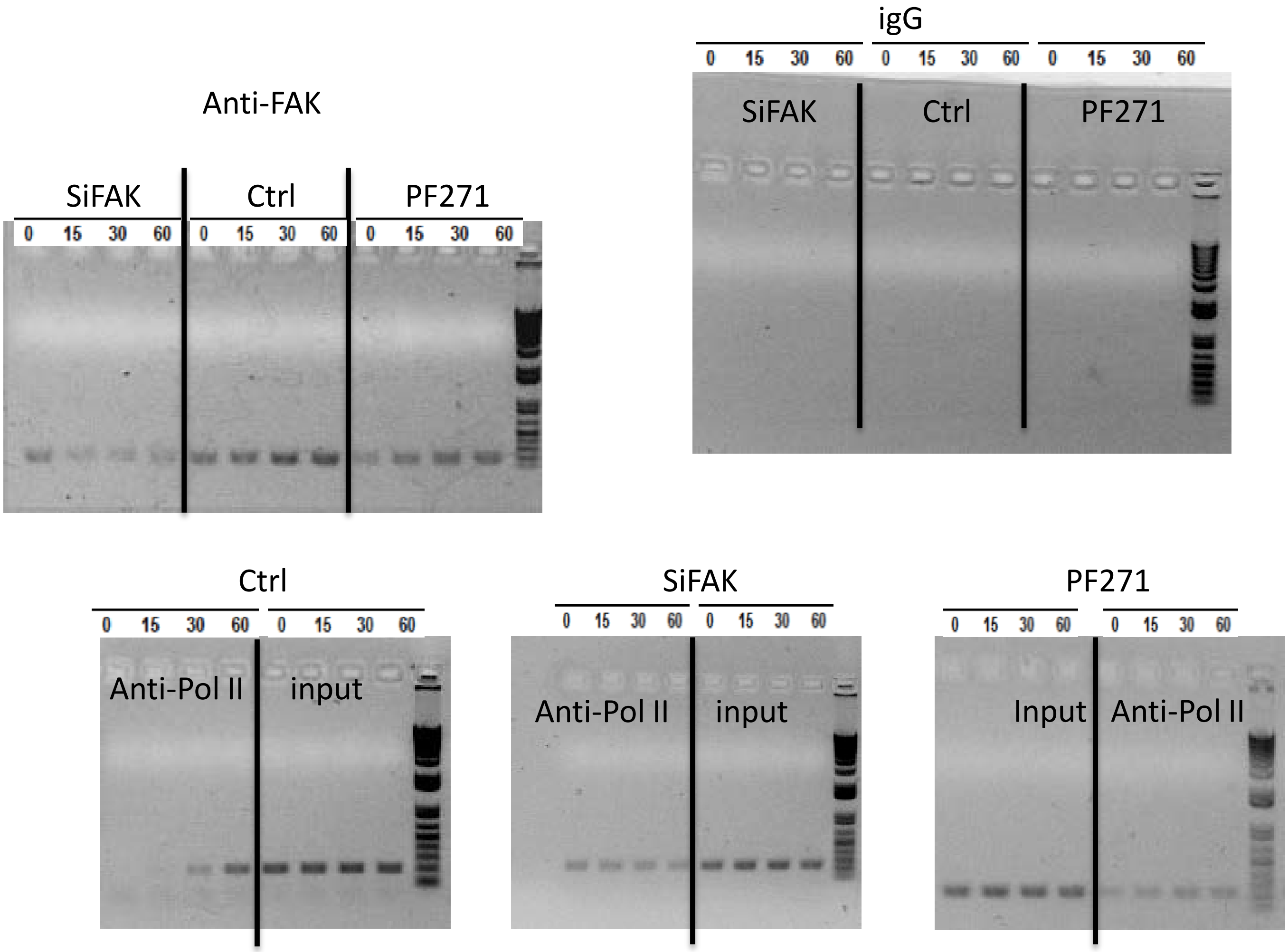

Supplement figure 11
